# Emerging Issues Regarding the Effects of the Microbiome on Lung Cancer Immunotherapy

**DOI:** 10.3390/biom15111525

**Published:** 2025-10-29

**Authors:** Kostas A. Papavassiliou, Amalia A. Sofianidi, Fotios G. Spiliopoulos, Angeliki Margoni, Athanasios G. Papavassiliou

**Affiliations:** 1 First University Department of Respiratory Medicine, ‘Sotiria’ Chest Hospital, Medical School, National and Kapodistrian University of Athens, 11527 Athens, Greece; 2 Department of Biological Chemistry, Medical School, National and Kapodistrian University of Athens, 11527 Athens, Greece; amaliasof@med.uoa.gr (A.A.S.); spiliopoulosfotis99@gmail.com (F.G.S.); angeliki.margoni@gmail.com (A.M.)

**Keywords:** lung cancer, microbiome, immunotherapy, immune checkpoint blockade, immune checkpoint inhibitors

## Abstract

Lung cancer remains the deadliest malignancy, with limited effective and long-term therapeutic options. Immunotherapy has revolutionized the therapeutic landscape of lung cancer. However, not everyone with lung cancer responds to immunotherapy, while, inpatients who temporarily derive clinical benefit, resistance eventually develops. The host microbiome has emerged as a pivotal player in cancer growth and progression. It has been implicated in the intricate connections between immune cells and tumor cells, ultimately augmenting immunotherapy efficacy in solid tumors, while simultaneously mitigating the immune-related adverse events associated with this type of treatment. Notably, lung cancer patients who obtain benefit from immunotherapy have been found to be colonized with specific bacterial populations, and it is this observation that the scientific community is trying to exploit, aspiring to improve lung cancer immunotherapy response rates. Delving deeper into the molecular mechanisms underpinning the effects of the microbiome on immunotherapy is an area that we should pay attention to if we want to utilize microbiome modulation in everyday clinical practice. Fecal microbiota transplantation, probiotics, targeted antibiotic interventions, and dietary modifications are among the strategies that are under investigation in clinical trials, with the ultimate endpoint of lengthening the life expectancy of lung cancer patients.

## 1. Introduction

Immunotherapy has transformed the therapeutic landscape for many solid malignancies. Exploiting innate immune mechanisms to attack tumor cells is a clever evolution in oncology, fostering the body’s natural “intelligence” [1]. In lung cancer, immunotherapy indications are diverse. Immunotherapy regimens including anti-programmed cell death protein 1 (PD-1) or anti-programmed cell death ligand 1 (PD-L1) agents are widely used in patients with resectable stage II-III non-small cell lung cancer (NSCLC) who do not harbor *epidermal growth factor receptor* (*EGFR*) or *anaplastic lymphoma kinase* (*ALK*) alterations, or patients with unresectable stage III *EGFR*-wild type NSCLC after chemoradiotherapy completion [2]. In metastatic NSCLC, anti-PD-1 or anti-PD-L1 agents are used as first-line treatment for patients without specific driver mutations [3,4]. Nonetheless, responses to immunotherapy are diverse, yielding only a 20–30% response rate in NSCLC [2,5]. Similar disappointing results are observed in small cell lung cancer (SCLC) [6], which is considered one of the most fatal solid cancers. The efficacy of immunotherapy in the treatment of SCLC is even poorer than that in NSCLC, remaining limited to first-line treatment of extensive-stage disease in conjunction with chemotherapy, with only moderate effects on life expectancy [7]. Even when immunotherapy is successful, its long-term benefits are restricted [8]. Improved strategies are urgently needed to ameliorate antitumor immune responses and enhance the immunotherapeutic armamentarium against lung cancer. Leveraging the human microbiome provides a vigorous approach in this direction. Indeed, the microbiome has been recognized as an emerging hallmark of cancer, as it is present not only systemically, but also within tumors [9]. This plethora of microorganisms creates ecosystems that reside within the human body––in the colon, on other mucosal surfaces, in organs, or in tumors themselves––affecting how tumors develop, progress, and respond to treatment [9,10]. Therefore, the interaction between the microbiome and immunotherapy in lung cancer and the underlying molecular mechanisms are a novel field of investigation.

## 2. Gut Microbiome Composition in Lung Cancer Immunotherapy Responders Versus Non-Responders

The host gut microbiota has been shown to regulate the tumor microenvironment (TME) by modulating immune responses and influencing reactions to anticancer therapeutic modalities [11]. Several immune cells are involved in this process, including T-helper 1 cells (Th1), CD8+ cytotoxic T-cells (CD8+ T-cells), macrophages, regulatory T-cells (Tregs), and dendritic cells (DCs) [12]. Notably, different gut microbiota compositions have been found amongst patients with lung cancer who respond and do not respond to immune checkpoint inhibitors (ICIs). In lung cancer, particularly in NSCLC, a more diverse gut microbiome has been correlated with ameliorated immune checkpoint blockade (ICB) therapy outcomes [13]. A recently published systematic literature review revealed a higher abundance of *Actinobacteria*, *Bacteroidetes*, and *Verrucomicrobiota* in patients with NSCLC who responded well to ICI treatment [14]. *Bifidobacterium*, a genus within the phylum *Actinobacteria*, has been preclinically found to promote DC function and trigger the accumulation of CD8^+^ T-cells in the TME [15]. This observation is further strengthened by another preclinical study, which demonstrated heightened immune responses against NSCLC induced by specific *Bifidobacteria* strains that enhanced the biogenesis of immune-stimulating molecules and metabolites [16]. Notably, the bacterium *Bifidobacterium longum* was found to be more common in lung cancer patients who responded to ICB [13]. However, controversial results were recently reported, indicating that abundant *Actinobacteria* in the gut microbiome of NSCLC patients was associated with shorter progression-free survival (PFS) [17]. *Alistipes putredinis* and *Parabacteroides distasonis*, both classified in the *Bacteroidetes* phylum, are enriched in patients with NSCLC who respond well to ICB [14]. The immunostimulatory effects of this phylum are mainly attributed to the activation of Toll-like receptor 4 (TLR4), which in turn potentiates transcription factors such as nuclear factor kappa B (NF-κB), thereby inducing the release of pro-inflammatory cytokines [18]. *Akkermansia municiphila*, a member of the *Verrucomicrobiota* phylum, has been also abundantly found in the feces of patients with NSCLC who exhibit an adequate response to PD-1 inhibition [19]. Aligned with the previous observations, a profusion of *Faecalibacterium* has been linked to increased immunotherapy efficacy in advanced NSCLC [20].

## 3. Molecular Mechanisms Engaged in the Interplay Between Lung Cancer Immunotherapy and Microbiome

Exploring further the interplay between lung cancer immunotherapy and the gut microbiome, the exact mechanisms driving this association are not fully understood. The available data to date derives only from preclinical observations. Gut microbiota ferment complex compounds found in dietary fibers produce metabolites such as short-chain fatty acids (SCFAs), which include butyrate, acetate, propionate, and vafetate [21]. The connection between SCFAs and ICI treatment has been recently confirmed by evaluating fecal and plasma SCFAs in patients with solid tumors [22]. Although the majority of patients were diagnosed with melanoma, patients with lung adenocarcinoma were also included [22]. These metabolites enter the bloodstream and have regulatory effects on immune cells, alongside tumor cells. For instance, the *Bifidobacteriaceae* family exploits butyrate to activate G protein-coupled receptor (GPCR) 43, also known as free fatty acid receptor 2 (FFAR2), in DCs. This leads to DC maturation, improved antigen presentation, secretion of interleukin-12 (IL-12), and increased antitumor activity of CD8+ T-cells, ultimately fostering the efficacy of anti-PD-1 therapy [23]. SCFAs can also induce epigenetic changes, impeding histone deacetylases (HDACs) [24]. How these changes impact on immune regulation and surveillance in lung cancer remains to be elucidated. Nevertheless, current data supports the application of HDAC inhibitors for enhanced immunotherapy outcomes [25,26].

SCFAs also generate energy for immune cells. They supply B-cells, memory T-cells, and effector T-cells with energy by regulating biochemical pathways such as glycolysis, the tricarboxylic acid (TCA) cycle, and β-oxidation to augment ICI efficacy [27]. Butyrate in particular promotes oxidative and glycolytic activity in memory T-cells [28]. Another gut microbiota-derived metabolite, tryptophan (Trp), has been shown to exert variable effects on immune cells, while its immunosuppressive actions are often highlighted [29]. Low levels of its metabolite, 3-hydroxyphthalate, were linked to prolonged median progression-free survival (PFS) in patients with NSCLC [30]. Although the exact mechanisms are not fully comprehended, there is room to suggest that immunosuppression attenuation plays a role in this relationship.

Intriguingly, *Bifidobacterium pseudocatenulatum* has been shown to secrete inosine (Ino), a purine metabolite that binds to the adenosine 2A receptor (A2AR), inducing Th1 differentiation and amplifying the impact of immunotherapy. The Ino-A2AR-cAMP-protein kinase A (PKA) signaling cascade is activated, contributing to the phosphorylation of cAMP response element-binding protein (CREB), which in turn upregulates the transcription of Il-12 receptor, beta 2 subunit (IL12Rβ2) and interferon γ (IFNγ), ultimately prompting Th1 differentiation and assemblage in the TME. While these observations have been made primarily in colorectal cancer (CRC) models and not in lung cancer models, several *Bifidocacteria* strains have been implicated in increased immunotherapy response rates in lung cancer as well [31]. Additionally, Ino could be a surrogate nutrient supply for CD8+ T-cells. It is well established that cancer cells possess reprogrammed metabolic pathways and have augmented metabolic demands, leading immune cells to starve. Ino provides an alternative carbon source for CD8+ T-cells; it is metabolized by T-cells into hypoxanthine (Hyp) and ribose-5-phosphate (R5P) through the enzyme purine nucleoside phosphorylase (PNP). Of note, the “ribosomal subunit” of Ino enters the central metabolic pathway, providing ATP and biosynthetic precursors for the glycolytic pathway and the pentose phosphate pathway (PPP) [32].

**Figure 1:**
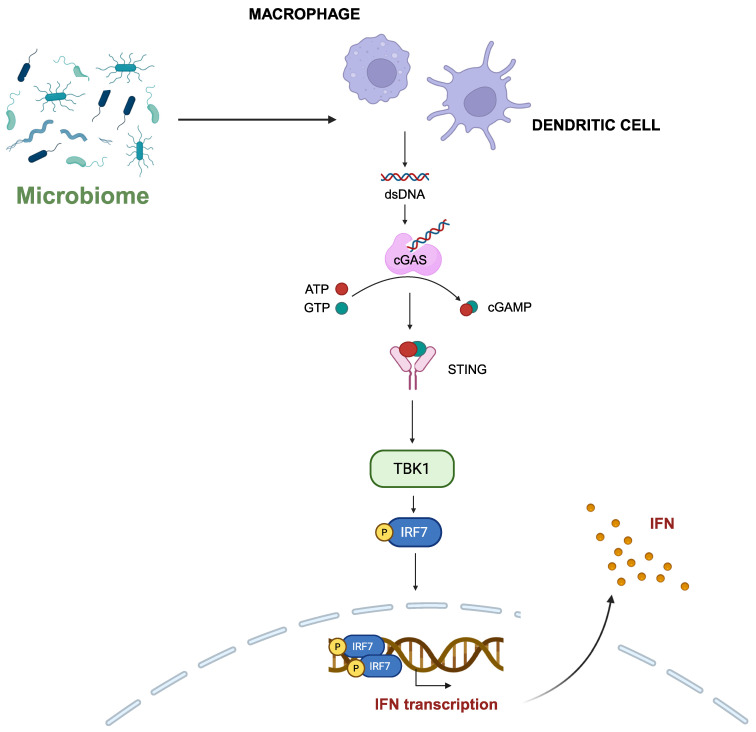
Cyclic GMP-AMP synthase (cGAS) senses double-stranded DNA (dsDNA) created by microbial-derived cyclic dinucleotides in the cytosol of macrophages and dendritic cells and synthesizes cGAMP. cGAMP, acting as the second messenger, activates cGAS-stimulator of interferon genes (STING), which is located in the endoplasmic reticulum (ER). STING then translocates from the ER to the Golgi apparatus, where it recruits TANK-binding kinase 1 (TBK1). The key substrate is interferon regulatory factor 7 (IRF7), which is phosphorylated and enters the nucleus to trigger type I IFN production, ultimately modulating the immune responses and improving immune checkpoint blockade (ICB) efficacy. Created using the tools provided by BioRender.com (accessed on 21 October 2025).

The cyclic GMP-AMP synthase (cGAS)-stimulator of interferon genes (STING) signaling axis has been implicated in the intricate connection between the gut microbiota and immunotherapy efficacy [33]. Microbial-derived cyclic dinucleotides (CDNs), such as cyclic diadenosine monophosphate (cdAMP), trigger activation of the STING pathway in macrophages, inducing the production of IFN-1 and the recruitment of immune cells to the TME [33]. Interestingly, several studies have examined oral administration of microbes in conjunction with anti-PD-1/PD-L1 antibodies to enhance antitumor effects [34]. Although this strategy will be discussed later in this article, oral administration of *Lactobacillus rhamnosus* GG (LGG) was investigated in combination with ICB in murine models of CRC and melanoma. LGG, part of the human gut microbiome, triggered IFN-1 production in DCs through the cGAS-STING-TANK-binding kinase 1 (TBK1)-IFN regulatory factor 7 (IRF7) axis, which is briefly described in Figure 1. Eventually, CD8+ T-cell cross priming is potentiated, improving the response to ICIs [35]. However, these observations have been made in murine models of CRC and melanoma and have not been validated in lung cancer models yet.

Programmed cell death ligand 2 (PD-L2) is another molecule that mediates immunotherapy responses induced by the host gut microbiome [36]. PD-L1 and PD-L2 both use PD-1 as a binding partner, but PD-L2 can also attach to repulsive guidance molecule b (RGMb), also known as DRG11-responsive axonal guidance and outgrowth of neurite (DRAGON) [37]. PD-L2 serves as a cancer cell immune escape mechanism [38]. It has been demonstrated that the gut microbiome downregulates PD-L2 expression and RGMb, stimulating antitumor immunity [36]. Certain gut bacteria abolish PD-L2 expression on DCs in lymphatic tissues, and DCs with low PD-L2 expression activate CD8+ T-cells to facilitate immune-mediated tumor destruction [36]. Blocking the PD-L2-RGMb pathway by antibodies in conjunction with anti-PD-1/anti-PD-L1 treatment promoted antitumor responses in mice that failed to respond to ICB monotherapy [36].

Apparently, deciphering the molecular mechanisms that support the promoting or even inhibitory effects of the microbiome on immunotherapy is an area in which we are falling behind. Most of the aforementioned observations are based on preclinical, rather than real-world, data and are not specifically derived from lung cancer models. More research in this field is anticipated in the coming years.

## 4. Microbiome-Centered Interventions to Improve Immunotherapy Responses in Lung Cancer

Employment of broad-spectrum antibiotics, repeated chemotherapy, and other therapeutic regimens disrupt gut microflora, restraining its tumor-fighting capacity. The intestinal microbiome could be manipulated with techniques that include fecal microbiota transplantation (FMT), probiotics, and dietary intervention. Actually, the enrichment of specific bacteria populations in the gut of immunotherapy responders suggests that strategies modulating microbiome diversity in cancer patients could yield promising antitumor results.

### 4.1. Fecal Microbiota Transplantation (FMT)

FMT is an innovative approach that could prove to be effective in expanding ICI efficacy [39]. ICI responder-derived FMT was explored in conjunction with PD-1 inhibition in patients with PD-1-refractory melanoma [40]. This successful phase II clinical trial paved the way for the clinical investigation of FMT in combination with immunotherapy in solid malignancies, not only for its anticancer responses, but also for the subsequent mitigation of the immune-related adverse events (irAEs) associated with immunotherapy [41]. Several early-stage clinical trials are ongoing in lung cancer [42], recruiting patients with distinct molecular profiles, such as NSCLC patients who do not harbor driver-gene mutations, to study the effects of FMT on immunotherapy efficacy [43]. Exploiting the microbiome diversity of this small proportion of NSCLC patients who respond to ICIs and further developing the technique of FMT could help increase immunotherapy response rates in lung cancer. It is a cost-effective method that does not require exquisite expertise and could be universally implemented, even in low-income countries. Whether FMT could also reduce the irAEs associated with ICB through modulation of host microflora is another area of future research [44]. It has been shown that *Bacteroidetes fragilis*, *Barnesiellaceae*, *Ikenellaceae*, *Bacteroidaceae* [45], and *Caloramator coolhaasii* [46] gut enrichment in patients with lung cancer correlates with decreased ICB toxicity. Similar observations have been made with *Bifidobacterium* and *Dialister* gut enrichment in patients with metastatic NSCLC [47]. Adverse events associated with FMT are usually mild, limited to gastrointestinal symptoms, such as bloating, flatulence, abdominal pain, and diarrhea. Less frequently, major complications such as bowel perforation, bacteremia, sepsis, and death due to multidrug-resistant pathogen transmission have also been described [48].

### 4.2. Probiotics

While both probiotics and FMT aim to restore balance within the gut microbiome, probiotics are over-the-counter drugs containing specific microbial populations [49]. FMT is a more targeted approach derived from patients with already displayed beneficial clinical outcomes, yet probiotics could be another successful method to deliver specific microbial strains with established immunotherapy benefit. Probiotic *Clostridium butyricum* MIYAIRI 588 strain therapy significantly improved PFS and overall survival (OS) in patients with NSCLC treated with ICB. Objective response rate (ORR) and disease control rate (DCR) were also ameliorated in lung cancer patients receiving probiotics alongside ICI therapy [50]. Remarkably, probiotic supplementation may also improve the declined efficacy of ICB during antibiotic treatment, which disrupts gut microflora composition [51]. Antibiotic use one month before or during ICI treatment has a negative impact on ICI efficacy [14]. The term antibiotic-immunotherapy exposure ratio (AIER) has been introduced, defined as “days of antibiotic/days of immunotherapy” during the whole period a patient receives ICB; notably, NSCLC patients with a higher AIER had shorter PFS and OS than those with low AIER [52]. However, medical oncologists are parsimonious when it comes to providing cancer patients with probiotics supplements as they can trigger active infection and subsequent bacteremia in immunocompromised subjects [53]. Minor adverse events of probiotic administration include, again, gastrointestinal symptoms, such as bloating and diarrhea. Safe manufacture of probiotics is also of utmost importance, in order to avoid administration of harmful pathogens [54]. The latest data is groundbreaking, rigorously supporting the use of probiotics in patients with solid tumors undergoing immunotherapy [14]. Probiotics are even easier than FMT to be launched and applied in the clinical setting. However, there are several limitations to this approach; diet is a confounding factor when it comes to probiotic administration. Dietary habits alter the microbiome composition of the gut, and, even if probiotics seem to improve immunotherapy efficacy in cancer patients, it might actually be diet promoting the effect [50].

### 4.3. Diet and Nutrition

There is growing evidence that diet can modify gut microbiome composition and, consequently, affect immune responses in the TME. Plant-based diets have the potential to augment ICI efficacy in NSCLC patients. It has been demonstrated that they boosted the abundance of *Actinobacteria*, *Bacteroidetes*, and *Verrucomicrobia*, simultaneously enhancing gut microbiome heterogeneity [14,53]. On the contrary, animal protein diets reduce the diversity of these microbiomes, hindering ICB [14]. Nevertheless, Gustafson et al. noted that patients who follow plant-centered diets tend to have a more healthy lifestyle, adopting exercise and physical activity in their everyday routine, strategies that improve overall health and can serve as converging factors in the previous observation [55]. The effects of different dietary compositions on microbiome diversity and immunotherapy effectiveness are another untrodden area; recent research unveiled that the ketogenic diet, a diet comprised of high-fat, adequate-protein, low-carbohydrate configurations, alters the epigenetic and immune environment of prostate cancer to defeat resistance to ICB remedies [56]. Long-term side effects of the ketogenic diet encompass hepatic steatosis, kidney stones, hypoproteinemia, and vitamin deficiencies [57]. A Mediterranean diet has been also proposed as a strategy to improve response to immunotherapy. As an established form of healthy eating, it has been shown to increase the efficacy of ICIs in advanced melanoma patients [58]. The outcomes of different dietary structures should also be investigated in lung cancer treatment. However, compliance with a specific type of diet is difficult, especially when it comes to cancer patients, where food aversion is common.

## 5. Conclusions—Outlook

**Figure 2:**
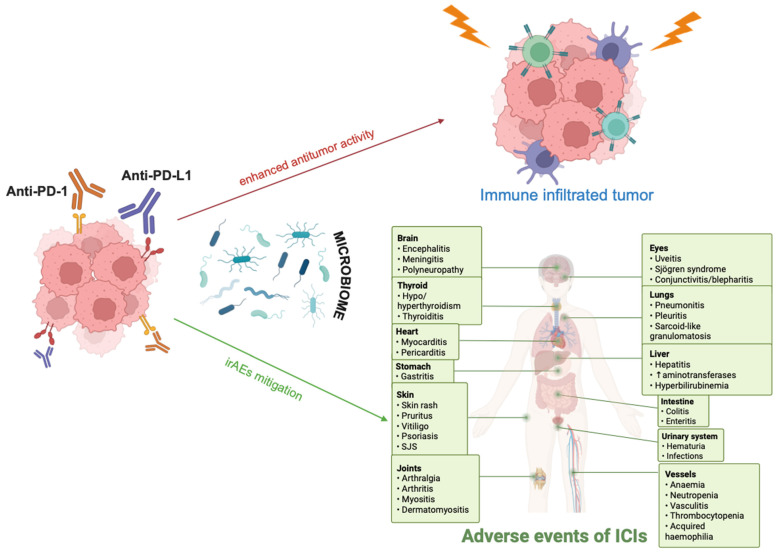
The microbiome intervenes in the immune checkpoint blockade (ICB), molding an immune tumor microenvironment (TME) that can enhance the antitumor effects of immunotherapy, while mitigating associated immune-related adverse events (irAEs). PD-1, programmed cell death protein 1; PD-L1, programmed cell death ligand 1; ICIs, immune checkpoint inhibitors; Microbiome, bacteria and various species of microorganisms [59]. Created using the tools provided by BioRender.com (accessed on 21 October 2025).

The microbiome is an easily modifiable target that could augment the antitumor potency of ICB and attenuate its toxicity (Figure 2).

Whereas intratumoral microbiomes are not effortlessly accessible, the gut microbiota is a reachable target. The gut microbiome can regulate the functions of immune cells, such as T-cells and B-cells, preserving immune homeostasis and refining the body’s tumor immunosurveillance. The gut microbiome holds the potential to be a novel biomarker for predicting sensitivity and adverse reactions related to immunotherapy in patients with lung cancer. Several signaling pathways have been proposed to be implicated in this interaction, with the STING signaling pathway being the most thoroughly explored. Microbiota-derived metabolites, such as SCFAs and amino acid metabolites, also modulate host innate immunity mechanisms and play a vital role in cancer growth and invasion. Nonetheless, further research is necessary in this field. Comprehending the molecular landscape behind microbiome and immunotherapy interconnection in lung cancer could help develop more targeted interventions.

Intriguingly, beyond bacteria, gut microbiota ecosystems are composed of several species of microorganisms, including protozoa, yeast, and viruses. Their contribution to immune homeostasis and their implication in TME behavior have not been studied to date. Future research should adopt a more holistic approach to the human microbiome located in different parts of the body and its role in tumorigenesis.

A forward-looking perspective embraces targeted antibiotic interventions to modulate the microbiome of lung cancer patients who do not obtain clinical benefit from immunotherapy. Illuminating the microbial composition of ICB responders could guide these interventions. Non-invasive fecal testing prior to ICI initiation could also predict which lung cancer patients are expected to respond to ICB. Various algorithms have already been launched, with the aim of implementation in clinical practice. FMT and probiotics have already been tested in the clinic, with favorable results so far. STING agonists could also be examined as a strategy to achieve maximum efficiency of ICB. The optimal composition of the gut microbiome remains largely unknown. Nonetheless, it should be highlighted that this approach has a major limitation; microbial composition is dynamic and can be independently modified by concurrent medications, dietary habits, and drug administration at any time during ICB. Forthcoming investigation should be directed to characterizing microbial signatures related to treatment success and evolving targeted microbiome modulation approaches to reinforce patient outcomes. More microbiome-centered clinical trials should be designed if we want to exploit human gut microbiota compositions for achieving better anti-lung cancer treatments.

## Data Availability

No new data were created or analyzed in this study.
